# Temporal Auditory Processing and Phonological Awareness in Children with Benign Epilepsy with Centrotemporal Spikes

**DOI:** 10.1155/2015/256340

**Published:** 2015-01-22

**Authors:** M. I. R. Amaral, R. L. Casali, M. Boscariol, L. L. Lunardi, M. M. Guerreiro, M. F. Colella-Santos

**Affiliations:** ^1^Child and Adolescent Health Program, Center for Investigation in Pediatrics, Faculty of Medical Sciences, State University of Campinas (FCM/UNICAMP), Tessália Vieira de Camargo 126, 13083-887 Campinas, SP, Brazil; ^2^Department of Neurology, Faculty of Medical Sciences, State University of Campinas (FCM/UNICAMP), Tessália Vieira de Camargo 126, 13083-887 Campinas, SP, Brazil; ^3^Department of Human Development and Rehabilitation (CEPRE), Faculty of Medical Sciences, State University of Campinas (FCM/UNICAMP), Rua Tessália Vieira de Camargo 126, 6111 Cidade Universitária “Zeferino Vaz”, 13083-887 Campinas, SP, Brazil

## Abstract

The aim of this research was to analyze temporal auditory processing and phonological awareness in school-age children with benign childhood epilepsy with centrotemporal spikes (BECTS). Patient group (GI) consisted of 13 children diagnosed with BECTS. Control group (GII) consisted of 17 healthy children. After neurological and peripheral audiological assessment, children underwent a behavioral auditory evaluation and phonological awareness assessment. The procedures applied were: Gaps-in-Noise test (GIN), Duration Pattern test, and Phonological Awareness test (PCF). Results were compared between the groups and a correlation analysis was performed between temporal tasks and phonological awareness performance. GII performed significantly better than the children with BECTS (GI) in both GIN and Duration Pattern test (*P* < 0.001). GI performed significantly worse in all of the 4 categories of phonological awareness assessed: syllabic (*P* = 0.001), phonemic (*P* = 0.006), rhyme (*P* = 0.015) and alliteration (*P* = 0.010). Statistical analysis showed a significant positive correlation between the phonological awareness assessment and Duration Pattern test (*P* < 0.001). From the analysis of the results, it was concluded that children with BECTS may have difficulties in temporal resolution, temporal ordering, and phonological awareness skills. A correlation was observed between auditory temporal processing and phonological awareness in the suited sample.

## 1. Introduction

(Central) auditory processing disorder [(C)APD] is defined as a deficit in neural processing of auditory information in the central nervous system. According to American Speech-Language Hearing Association (ASHA) [1], (C)APD may coexist with other disorders and may be associated with difficulties in higher order language, learning, and communication functions. It has been recognized that patients with epilepsy may present speech recognition impairments or speech processing difficulty [2], suggesting a functional deficit in central auditory processing. One of the most common forms of epilepsy is benign childhood epilepsy with centrotemporal spikes (BECTS), also known as rolandic epilepsy.

BECTS is an electroclinical syndrome [3] characterized as focal, genetically determined, and age-dependent. Patients usually present predominantly nocturnal seizures with unilateral or bilateral centrotemporal dysphasic spikes waves. Simple febrile seizures may occur before rolandic seizures in approximately 10 to 20% of cases. Seizures typically begin between 3 and 12 years and resolve spontaneously until 15–18 years. It is usually diagnosed by the association of clinical findings and the electroencephalogram (EEG), which has a normal background cerebral activity with high voltage sharp waves in the centrotemporal (rolandic) region, followed by slow waves activated by sleep [4].

BECTS was always considered a benign developmental disorder, because of the absence of obvious anatomic lesions, predictable spontaneous remission of seizures, and evidences of no cognitive and language impairments when compared to normal children [5, 6].

However, in the last 10 years, the “benign” term has been questioned. Studies have shown some degrees of cognitive deficits during the active epileptic phase [7–9]. Neuropsychological alterations have been described for language [10–13], impairment in verbal and attention skills [14, 15], executive functions and memory [16–18], and phonological awareness [19, 20].

Other studies also reported difficulty to process speech in the presence of background noise, even with normal hearing [21], worse performance on dichotic listening compared to controls [22, 23], and evidences of cortical auditory dysfunction based on electrophysiological measures [24, 25].

Despite the evidences, the correlation of language deficits, worsening academic performance, and auditory processing in BECTS has not been totally explored. The proximity of rolandic and perirolandic regions with supratemporal plane of the primary auditory cortex (Heschl's gyrus) supports the hypothesis that electrical discharges in these areas may result in impairment of cortical auditory function if there is a deficit in the central auditory nervous system (CANS) [22].

To our knowledge, no studies were made about temporal auditory processing and its relationship with phonological awareness in a Brazilian pediatric population with BECTS. Temporal auditory processing (TAP) is defined as the perception of sound or the alteration of durational characteristics within a restricted time interval. Some authors argued that TAP may be the underlying component of many auditory processing abilities, including the processing of both verbal and nonverbal acoustic signals such as speech perception in quiet and in background noise, localization, discrimination, binaural integration, and binaural separation [26–28].

Among temporal auditory skills which can be assessed by behavioral tests, there are temporal resolution and temporal ordering. Such abilities require an accurate processing of the sound time structure of the signal, time and order of occurrence. Those abilities contribute to identifying small phonetic elements in speech, important cues that help speech perception. Difficulties found in these skills suggest interference in normal speech perception and phonemes recognition.

Phonological awareness is a metalinguistic skill. It refers to the ability to analyze and manipulate the component sounds of words, including syllables and phonemes. It also refers to the awareness that linguistic units are repeated in different words and that they have a direct relation to orality [29]. It has been shown to be a primary factor underlying early reading achievement and deficits in phonological awareness have been linked to reading [30].

Based on the hypothesis that the accuracy to differentiate small characteristics of speech sounds provided by temporal auditory skills is directly related to phonological awareness, the aim of this research is to evaluate temporal auditory processing and phonological awareness in school-age children with BECTS.

## 2. Methods

### 2.1. Study Design

This is a prospective cross-sectional study conducted at the Laboratory of Audiology from Center of Studies and Researches in Rehabilitation (CEPRE) and Neuroepilepsy Ambulatory Unit/University Hospital, both from the State University of Campinas (Unicamp/Brazil), after its approval by the Ethics Committee (protocol 254/2010). Written informed consent was obtained for all participants.

Eligibility criteria included children from public elementary school, aged between 8 and 15 years old, right handedness, normal findings on otoscopy exam, average of pure tone threshold within ≤15 dB of hearing level at frequencies of 500 Hz, 1, 2, and 4 Khz, and “A” type tympanogram [31].

Thirty school-age children ranging from 8 to 15 years old participated in the study. The patient group (GI) consisted of 13 children diagnosed with BECTS. The control group (GII) consisted of 17 healthy children.

### 2.2. Study Subjects

#### 2.2.1. Patient Group—GI

All patients had been diagnosed with BECTS on the basis of a clinical history of seizures and EEG. Children whose epilepsy had the well-defined clinical and electrophysiological features of the typical syndrome [3] were included in the cohort. Other inclusion criteria for GI were as follows:

- normal neurological examination performed by neurologist;
- normal brain MRI performed by a specialist through the equipment Elscint Prestige 2.0 T with posterior multiplanar reconstruction and curvilinear reformatting in 3D magnetic resonance imaging;
- normal intelligence quotient (IQ) according to the Wechsler Intelligence Scale for Children-WISC-III (IQ > 80) [32].

Parents were asked about the occurrence of auditory complaints, educational difficulties and historical of recurrent otitis media during the first years of life. The hearing complaints raised included the following: difficulty of hearing in quiet and in noise, tinnitus, constant need to repeat information, and difficulty in sound localization and in understanding oral instructions. The aspects of school difficulties included questions about the performance in reading, writing, and mathematics, letter exchanges in speech and writing, low academic performance at school, and history of grade repetition.

Four children were excluded based on the inclusion/exclusion criteria and 13 children (8 males) with BECTS participated in this study.

#### 2.2.2. Control Group—GII

The normal control sample included children with no history suggestive of pathologies involving the central nervous system (CNS) and no history of delay or abnormality in language and learning. Children were selected from a public elementary school in our city. The inclusion criteria also included good academic performance reported by the responsible teacher through a questionnaire and no hearing complaints or difficulties reported by teacher and parents. Based on these criteria, 17 healthy children (8 males) participated in this study.

### 2.3. The Study Procedures

After the selection of subjects, the following procedures were performed: assessment of temporal auditory processing, composed of the Gaps-in-Noise test (GIN) [27] and Duration Pattern test (DPT) [33] and Phonological Awareness test (PCF) [34]. Auditory tests were applied through an* Interacoustics* AC40 audiometer with a* Phillips* CD recorder, and the tests were carried out in a sound-treated double room presented at 40 dBSL (sensation level), based on the average of pure tone thresholds at 500, 1000, and 2000 Hz. The tests were not performed by blinded examiners.

The GIN test assesses auditory temporal resolution. The test materials consist of series of 6-second white noise stimuli, separated from each other by a 5-second silent interval, in which 0 to 3 silence gaps of different durations (2, 3, 4, 5, 6, 8, 10, 12, 15, 20 ms) were embedded within each segment. The occurrence of gap duration and location within noise segments are pseudorandomized. There are also no gaps between a few segments. Every gap appears six times on each list, totaling 60 gaps per ear. The test material was presented to each ear, separately. The task required was raising hand whenever they were able to identify the silence gaps, in milliseconds (ms). Two types of scores were calculated: percentage of correct identification (PCI) and the gap detection threshold (GDT). The GDT was defined as the shortest gap duration that the subject was able to identify at least four out of six times. The PCI was the number of correct responses divided by the total number of gaps × 100.

The Duration Pattern test (DPT) assesses the ability of temporal ordering. The frequency of tones is held constant at 1000 Hz with two 300 ms intertone intervals, and duration of tones is the factor to be identified. Short-S (250 ms) and long-L (500 ms) pure tones were presented in six possible combinations of a three-tone sequence (LLS, LSL, LSS, SLS, SLL, and SSL). Thirty sequences were applied in two modalities to each ear: a verbal description of the sequence (naming) and humming. The score calculated was the percentage of correct responses.

Phonological Awareness test (PCF) was used for the assessment of phonological awareness. All children were investigated by an experienced speech and language therapist. The PCF has a normative data in Portuguese; it was developed based on the Sound Linkage [35]. The test presents ten subtests: syllable synthesis, phonemic synthesis, syllable segmentation, phonemic segmentation, syllable manipulation, phonemic manipulation, syllable transposition, phonemic transposition, rhyme, and alliteration. Each subtest is composed of two initial items for practice and four items of test. In order to improve analysis, the subtests were grouped into 4 categories: syllabic, phonemic, rhyme, and alliteration. The outcomes for children in PCF were presented as score, and the maximum was four points per subtest and forty points in total.

All results obtained were recorded in a computerized database and tables of the results were constructed. Statistical analyses were conducted using the Statistical Package for the Social Science (SPSS) version 17. The gender homogeneity of GI and GII was tested by the distribution of relative frequency (percentage) using the test of Equality of Two Proportions. Student's* t*-test was used to compare scores from right and left ears. Mean, median, and standard deviation for individual procedures were calculated separately for GI and GII. ANOVA was used to compare patient group with controls. Pearson correlation was used to investigate the correlation between phonological awareness tasks and temporal auditory tests. The significance level was set to 0.05 and data in which statistically significant differences were found are highlighted in bold.

## 3. Results

The sample consisted of 30 school children aged 8 to 15 years. In GI (*N* = 13), ages ranged from 9.6 to 14.11 years (mean 11.6 ± 1.8; 8 males) and GII (*N* = 17) was from 8.2 to 14.4 years (mean 10.6 ± 1.9; 8 males). The groups were homogeneous in age (*P* = 0.169) and gender (*P* = 0.431).

Demographic data of patients from GI and the presence or absence of auditory and learning complaints are summarized in Table 1.

Gap detection threshold (GDT) results were similar for both ears in GI and GII (*P* = 0.179 and *P* = 0.163, resp.) and also percentage of correct identification (PCI) (*P* = 0.095 and *P* = 0.275). The same similar performance between right and left ears occurred in Duration Pattern test, in GI and GII, and in both modalities: naming (*P* = 0.069 and *P* = 0.462) and humming (*P* = 0.611 and 0.245). Since no significant difference was found in gender distribution and performance of right and left ears, the results were amalgamated in the calculations.

The mean GDT in GI [8.2 (+2.1) ms] was found to be higher (worst) when compared to control group [4.5 (+0.9) ms]. Similarly, the PCI was found to be lower [59.6 (+11.6)%] when compared to GII [79.0 (+7.0)%]. These differences were statistically significant, as shown in Table 2.

Regarding Duration Pattern test, the results indicate a statistically significant worse performance of GI when compared to GII, both in naming and humming modalities, as shown in Table 3.

Phonological Awareness test (PCF) results showed a statistically significant mean difference between GI and GII for all evaluated categories. GI presented a worse performance when compared to controls. Mean, median, and standard deviation are summarized in Table 4.

Statistical analysis revealed significant correlation between temporal auditory tests and phonological awareness skills assessed. The results from GI and GII are shown in Tables 5 and 6, respectively.

## 4. Discussion

Disorders involving perceptual processing of auditory information by the central auditory nervous system (CANS) may present themselves isolated or comorbid with developmental disorders and/or neurological conditions, such as benign epilepsy of childhood with centrotemporal spikes (BECTS). This study aimed to analyze the temporal auditory processing and phonological awareness in a sample of children diagnosed with BECTS.

According to parents' reports in GI, 7 of 13 children (53.8%) presented some kind of difficulty and/or hearing complaints, and only 1 child (7.7%) had a history of recurrent otitis media in childhood and use of ventilation tube (Table 1). Among the reported auditory complaints, we highlight the hearing difficulty in the presence of background noise, often requiring the information to be repeated, and difficulty understanding oral instructions. Regarding academic complaints, 10 of 13 children (76.9%) had learning difficulties and worse academic performance compared to their colleagues according to parent's report, especially in reading and writing skills. These results corroborate studies that have reported specific learning disabilities and decreased academic performance in children with BECTS, which are demonstrated by tests and also by parents and teacher's statement of academic underachievement [36, 37].

Temporal resolution ability involves the shortest duration of time which an individual can discriminate between two signals. It can be assessed using a variety of different procedures and GIN test is described as a procedure with good specificity [38].

To differentiate the normal versus abnormal scores, two standard deviations (SD) from the mean of the control group were considered. Accordingly, it was 6.3 ms for GDT and 65% for PCI. The scores were considered abnormal in GDT when the mean was above (higher threshold) 6 ms and in PCI when it was below (lower identification) 65%. These values are consistent with other studies that evaluated the pediatric population, without hearing and learning disorders, through GIN [39–41]. With reference to this criterion, it was found that 12 out of 13 patients (92.3%) from GI demonstrated abnormal scores for GDT (above 6 ms), and 10 out of 13 patients (76.9%) had abnormal scores for PCI, indicating deficit in temporal resolution ability.

Studies point to a correlation between the performance in gap detection tasks and skills involving perception and discrimination of speech, in silence and in noise [42, 43]. Difficulties perceiving rapid changes in the acoustic signal influence phoneme identification and aspects related to speech recognition. Detection of a silence gap embedded in noise requires the fine processing of temporal structures of sound and deficits in temporal resolution have been associated with impairments in learning, reading, and phonological processing [44].

Processing of temporal structures of sound is dependent on the integral auditory system for perfect transmission of acoustic information through auditory pathway, but studies show evidence that tasks involving detection of gap seem to be more sensitive to cortical lesions (primary auditory cortex) as opposed to brainstem involvement [45]. In a study with 26 patients with refractory complex partial seizures and mesial temporal sclerosis (MTS), temporal resolution was assessed through GIN test and results were compared to 50 normal controls. The results indicated worse GDT and PCI in MTS patients compared to controls. The authors discussed that the effect of MTS on central nervous system possibly makes it vulnerable to temporal processing deficits and GIN test is sensitive to cortical lesions [46].

Regarding Duration Pattern test, GI presented statistically lower scores when compared to GII (Table 3). The two SD scores were 62.1% and 65.5%, respectively, and 9/13 (69.2%) demonstrated abnormal scores for naming and 7/13 (53.8%) for humming.

Temporal ordering refers to the perception and processing of two or more auditory stimuli in their order of occurrence in time and involves inter- and intrahemispheric areas. The ability to recognize, identify, and sequence auditory patterns involves several processes which require information integration from both hemispheres across corpus callosum [47]. It is further known that more global perceptual-cognitive abilities are also involved and should be considered, such as attention and memory [48].

The type of response required by DPT requires the individuals to memorize the association between the name (long or short) and the specific sound, ensuring the correct nomination, while they must also memorize the whole sequence so that the sounds are ordered correctly. In this process, both cerebral hemispheres are involved, and the recognition of the acoustic contour of the auditory stimulus is processed by the right hemisphere and transferred via the corpus callosum to the left hemisphere. Dominant for language in most right-handed individuals, the left hemisphere is responsible for serial ordering of temporal information and linguistic nomination. It is understood, therefore, that there is an interhemispheric interaction in the temporal ordering, even if the sequence of the stimulus is not constituted by linguistic elements (verbal) [26].

Boatman et al. [25] evaluated fourteen patients (7 with BECTS and 7 age- and gender-matched controls) using behavioral and electrophysiological methods to study multiple auditory functions. Results of behavioral assessment pointed to the fact that all seven BECTS patients were impaired on at least one of the eleven tests used to assess auditory function. Unlike our findings, only two patients had difficulty with tone (pitch) sequencing and their performance was not significantly worse than controls. The discrepancy in findings may be due to possible differences in sample size and test parameters such as mode of application and task required.

In agreement with the findings of this current study, some researchers have pointed to evidences of auditory dysfunction in BECTS, which could reflect specific dysfunctions of primary and nonprimary areas of the auditory cortex in the temporal lobe caused by rolandic discharges that occur in the centrotemporal region and adjacent areas, such as perisylvian and temporoparietal regions [22, 24, 25].

Boatman et al. [25] confirmed their findings by associating behavioral and electrophysiological evaluation. Data show abnormal results in the group of children with BECTS on cognitive potentials, P300 mismatch negativity (MMN). Liasis et al. [24] evaluated the auditory function of 12 children with BECTS with auditory event-related potentials. Based on the deficits found, the authors suggest that it is possible that more than one auditory process is implicated in some language difficulties previously described in this population. From the results of our research, the auditory temporal processing should be considered as one of the fundamental processes that contribute to the understanding of speech information and it can be found altered in children with BECTS.

The results of Phonological Awareness test (PCF) point to specific deficits of children from GI when compared to controls subjects, which demonstrate difficulty in analyzing and manipulating the component sounds of words (Table 4). These finding are consistent with previous studies pointing evidence for phonological awareness difficulties in BECTS children, which impact on their level of reading and writing [16, 19]. Northcott et al. [19], in order to delineate the memory and phonological awareness profile of children with BECTS, evaluated 42 children and compared results to a proper control group. Five phonological awareness abilities were assessed (QUIL test) [49]: nonword spelling, nonword reading, visual rhyme detection, phoneme detection, and phoneme manipulation. The results pointed to a significantly better performance in control group in 2 of the 5 subtests, nonword reading and visual rhyme detection.

In an intratest analysis, both in GI and GII, lower scores and higher variability of responses in tasks involving phonemic awareness can be observed when compared to tasks involving syllabic awareness. These results were expected, since the development of phonological awareness involves hierarchical patterns of complexity and syllable awareness is more easily acquired than phonemic awareness [50]. Other tasks, such as rhyme and alliteration, also require the individual to be able to perceive acoustic changes that occur rapidly (milliseconds), adequate perception of the order of presentation of linguistic stimuli and acoustic contour of sound, allowing the analysis of similarities and differences between syllables and phonemes [51]. Therefore, deficits in perceiving rapid changes in the acoustic signal and their order of occurrence influence not only the perception of the phoneme (segmental level) but also suprasegmental aspects of speech recognition, such as intonation and rhythm (prosodic level) [52].

Specific deficits in perception and process of short acoustical elements and their order of occurrence have been associated in literature with impairments in language, reading, writing, and phonological processing [53]. According to Soares et al. [54], difficulties to process stimuli that incorporate acoustic cues of short duration and sequence, as the speech is structured, may precede and predict subsequent disorders in phonological representation that have been found in children with impairments in language, reading, and writing.

In our sample, Pearson's correlation showed different results in GI and GII. Our data show, in GI, a very strong to moderate positive relationship between all categories of PCF and DPT (naming and humming), and there was no statistically significant correlation between PCF and GIN (Table 5). Results from GII (controls) point to strong negative relationship between the gap detection threshold from GIN and the PCF (total score and phonemic task) as well as strong positive relationship between the percentage of correct identifications in GIN and the same categories of PCF (total score and phonemic task) (Table 6).

These correlations found in GI and GII suggest that auditory processing disorders may lead to a poor performance in phonological awareness skills in children with BECTS. This result corroborates with studies that have showed correlation between temporal auditory skills and phonological processing [51, 54]. From these results, we believe that the temporal auditory processing deficits found in children of this sample could be related to difficulties in speech discrimination and phonological processing in this pediatric population, as previously reported in the literature [11, 19, 25].

The results of Pearson's correlation in control group (GII) are in agreement with recent evidence that temporal resolution is a critical auditory skill necessary in accurate auditory processing of sound information. Learning difficulties in oral language are attributed in part to an inability of processing rapidly changing acoustic cues in the speech sequence, as well as difficulties in phonological discrimination. These tasks are related to the temporal resolution that could predict performance on tasks of reading, writing, and spelling [41]. However, the results from GI point to the fact that, for this study sample, temporal resolution may not be the skill that plays the most important role in performing tasks involving phonological awareness.

It is known that the recognition and identification of acoustic patterns, assessed by the temporal ordering tasks, involve a variety of cognitive and perceptual processes, and the temporal ordering is an ability that also plays a fundamental role for the correct speech perception, especially in relation to the perception of suprasegmental aspects of speech [55]. It was possible to observe a statistical relationship between a low performance in temporal ordering ability and all categories of PCF, in GI. These results point to the hypothesis that difficulties in temporal ordering ability seem to influence more failures in phonological skills in the studied sample when compared to temporal resolution ability.

It also stands out that the temporal ordering task can be considered auditory and cognitively more complex than gap detection tasks. Therefore, DPT may represent a higher degree of difficulty for children with BECTS considering evidence pointing to the existence of language impairment in this population due to the overlap of cortical language areas with epileptic activity [10]. On the GIN test, the response required is exclusively motor and totally nonverbal, whereas the temporal ordering requires a verbal answer of the sequence (naming), beyond the involvement of other cognitive processes, such as short-term memory, in both modalities of response to the test.

Monjauze et al. [10] claim that BECTS is a suitable model to investigate the links between epileptic activity and language, and the localization of the epileptic focus in the perisylvian language areas would appear to suggest specific impairments of this function. Due to the complexity of the relationship among epilepsy, language, and cognitive and auditory functions, it is difficult to distinguish the direct effects related to the epileptic discharges in language, such as phonological awareness, from other possible related factors, such as temporal auditory deficits. However, there is scientific evidence that regions involved in phonological processing are also activated in temporal auditory processing tasks, suggesting that phonological processing and auditory processing are closely related [49].

This study has some limitations. Because of the reduced number of subjects, some characteristics of seizure that can act as major generators of cognitive disorders were not explored, such as age at onset of seizures, use of antiepileptic drugs (AEDs), seizure frequency and duration, and the duration of the disease. More studies are necessary in order to understand if those multiple factors can influence auditory processing performance in BECTS.

In conclusion, the results of our study, combined with evidences that areas involving phonological processing are also activated in auditory temporal processing tasks [49], support the hypothesis that these processes are closely related and may find themselves altered in children with BECTS. Despite the spontaneous remission of seizures, BECTS is a common electroclinical syndrome, and a longitudinal follow-up is important, especially regarding the academic performance of these children. Formal assessments should be performed whenever necessary, especially regarding aspects of reading, writing, memory, and auditory processing. Appropriate intervention with training of temporal auditory and phonological processing skills can result in better academic performance of children with BECTS.

## 5. Conclusion

From the analysis of the results, the following was concluded.

- Children from GI presented statistically worse performance on temporal auditory processing assessment when compared to GII (*P* < 0.001).
- 12 of 13 children from GI (92.3%) demonstrated abnormal results for gap detection threshold (above 6 ms).
- 9 of 13 children from GI (69.2%) demonstrated abnormal results from Duration Pattern test in a verbal description of the sequence (naming).
- Children from GI performed significantly worse in all of the 4 categories of Phonological Awareness test: syllabic (*P* = 0.001), phonemic (*P* = 0.006), rhyme (*P* = 0.015), and alliteration(*P* = 0.010).
- Statistical analysis showed a significant positive correlation between the Phonological Awareness test and Duration Pattern test, in GI (*P* < 0.001).

## Figures and Tables

**Table 1:** Demographic data of patients with BECTS (GI).

Case	Age^*^	Gender	Age of first seizure	Age of last seizure	AED^**^	Auditory complaints	History of otitis media	Learning difficulties
1	9.6	M	5.7	7.7	Withdrawal	Yes	No	Yes
2	9.8	F	2.6	7.4	Yes	Yes	No	Yes
3	9.8	F	4	9.4	Yes	No	No	Yes
4	9.10	M	8.10	9.6	Yes	No	No	Yes
5	10.8	M	8.4	10.1	Yes	No	No	Yes
6	11.5	F	1.7	6.4	Withdrawal	Yes	No	Yes
7	11.6	M	1.6	5.8	No	No	No	Yes
8	11.9	M	1.6	8.9	Yes	Yes	No	Yes
9	12	F	8.7	9.4	No	No	No	No
10	12.3	F	1.4	6	Yes	Yes	No	Yes
11	13.11	M	0.9	4	No	Yes	Yes	No
12	14	M	4.7	6.7	No	No	No	No
13	14.11	M	9	14.9	Withdrawal	Yes	No	Yes

Mean	11.6		4.5	8.1	—	—	—	—

(±SD)	1.8		3.2	2.8	—	—	—	—

^*^Years.month.

^**^AED = antiepileptic drug's on evaluation data.

**Table 2:** Performance of children from GI and GII on Gaps-in-Noise (GIN) test.

GIN	GDT (ms)		PCI	
	GI	GII	GI	GII
*N*	26	34	26	34
Mean	8,2	4,5	59,6	79,0
Median	8,0	4,0	58,3	80,0
SD	2,1	0,9	11,6	7,0
*P* value	**<0,001**		**<0,001**	

**Table 3:** Performance of children from GI and GII on Duration Pattern (DPT) test.

DPT	Naming (%)		Humming (%)	
	GI	GII	GI	GII
*N*	26	34	26	34
Mean	53,3	83,7	62,1	87,9
Median	51,7	85,0	55,0	90,0
SD	26,3	10,8	23,8	11,2
*P* value	**<0,001**		**<0,001**	

**Table 4:** Performance of children from GI and GII on Phonological Awareness test (PCF).

PCF		*N*	Media	Median	SD	*P* value
PCF-total	GI	13	31,9	34,0	7,0	**0,002**
	GII	17	37,8	38,0	1,8	
Syllabic	GI	13	14,5	15,0	1,8	**0,011**
	GII	17	15,8	16,0	0,6	
Phonemic	GI	13	10,9	12,0	4,0	**0,006**
	GII	17	14,2	14,0	1,7	
Rhyme	GI	13	3,0	4,0	1,4	**0,015**
	GII	17	3,9	4,0	0,3	
Alliteration	GI	13	3,5	4,0	0,7	**0,010**
	GII	17	3,9	4,0	0,2	

**Table 5:** Correlation between temporal auditory tests and phonological awareness in children from GI.

GI	PCF-total		Syllabic		Phonemic		Rhyme		Alliteration	
	Corr (*r*)	*P* value	Corr (*r*)	*P* value	Corr (*r*)	*P* value	Corr (*r*)	*P* value	Corr (*r*)	*P* value
DPT										
DPT-N	71.6%	**<0,001** ^*^	53.9%	**0,004** ^*^	74.10%	**<0,001** ^*^	58,90%	**0,002** ^*^	39,20%	**0,047** ^*^
DPT-H	68,50%	**<0,001** ^*^	53,40%	**0,005** ^*^	69,30%	**<0,001** ^*^	57,00%	**0,002** ^*^	41,20%	**0,036** ^*^

GIN										
GDT	−30,0%	0,136	−35,0%	0,08	−29,3%	0,147	−23,0%	0,258	2,00%	0,921
PCI	7,10%	0,731	26,30%	0,193	0,50%	0,982	6,50%	0,751	−11,6%	0,571

DPT: Duration Pattern test; DPT-N: naming; DPT-H: humming.

GDT: gap detection threshold; PCI: percentage of correct identifications.

**Table 6:** Correlation between temporal auditory tests and phonological awareness in children from GII.

GII	PCF-total		Syllabic		Phonemic		Rhyme		Alliteration	
	Corr (*r*)	*P* value	Corr (*r*)	*P* value	Corr (*r*)	*P* value	Corr (*r*)	*P* value	Corr (*r*)	*P* value
DPT										
DPT-N	26.7%	0.126	11.7%	0.510	19.9%	0.338	33.5%	0.053	8.8%	0.622
DPT-H	0.1%	0.997	−5.0%	0.779	0.0%	1.000	4.1%	0.820	6.5%	0.715

GIN										
GDT	−44.8%	**0.008** ^*^	7.5%	0.671	−52.8%	**0.001** ^*^	11.6%	0.515	0.8%	0.963
PCI	50.3%	**0.002** ^*^	−6.5%	0.713	57.5%	**<0.001** ^*^	−16.5%	0.352	−11.3%	0.524

DPT: Duration Pattern test; DPT-N: naming; DPT-H: humming.

GDT: gap detection threshold; PCI: percentage of correct identifications.
